# Partners in crime: the TGFβ and MAPK pathways in cancer progression

**DOI:** 10.1186/2045-3701-1-42

**Published:** 2011-12-28

**Authors:** Douglas A Chapnick, Lisa Warner, Jennifer Bernet, Timsi Rao, Xuedong Liu

**Affiliations:** 1 Department of Chemistry and Biochemistry and Molecular, Cellular and Developmental Biology; 2 University of Colorado, Boulder, Colorado 80309, USA

**Keywords:** TGFβ, Metastasis, ERK, MEK, Ras, Crosstalk, Signal Transduction

## Abstract

The TGFβ and Ras-MAPK pathways play critical roles in cell development and cell cycle regulation, as well as in tumor formation and metastasis. In the absence of cellular transformation, these pathways operate in opposition to one another, where TGFβ maintains an undifferentiated cell state and suppresses proliferation, while Ras-MAPK pathways promote proliferation, survival and differentiation. However, in colorectal and pancreatic cancers, the opposing pathways' mechanisms are simultaneously activated in order to promote cancer progression and metastasis. Here, we highlight the roles of the TGFβ and Ras-MAPK pathways in normal and malignant states, and provide an explanation for how the concomitant activation of these pathways drives tumor biology. Finally, we survey potential therapeutic targets in these pathways.

## Introduction

A cell must acquire several key characteristics in order to become cancerous: proliferation without limit in the absence of extracellular signals, resistance to apoptosis, evasion of anti-growth signals and immune destruction, as well as increased cellular motility [1,2]. The TGFβ and Ras-MAPK pathways have each been implicated in all of the cellular processes that a cancer cell must exploit on the path to malignancy. However, it is becoming increasingly clear that these pathways interact, such that the resulting signal crosstalk contributes largely to the acquisition of many of the key characteristics of a cancer cell.

The TGFβ signaling pathway regulates differentiation, migration, and death during normal development. Mutations to the TGFβ signaling pathway are commonly seen in many genetic diseases and cancers [3-5]. For example, early lesions in colorectal and pancreatic cancers frequently include mutations of the cell surface TGFβ receptors or Smad transcription factors. However, the role of the TGFβ ligand in cancer progression has been somewhat puzzling and paradoxical due to its multiple, often opposing, effects on cell growth. This TGFβ paradox is best exemplified by the fact that TGFβ has been shown to act not only as a tumor suppressor, but also as a promoter of tumor growth and metastasis [6,7].

Ras-MAPK signaling has been linked to fundamental cell processes such as differentiation, migration and proliferation [8,9]. The GTPase Ras and the Ras-MAPK cell surface receptors that initiate the intracellular signaling pathway (EGFR, FGFR, etc.) are often mutated in colorectal and pancreatic cancer, and these mutations lead to a constitutively active Ras-MAPK pathway [10]. Once commandeered, MAPK transcription factor substrates promote unchecked cellular proliferation, leading to tumor initiation. This review will examine the parallels between and intersections of these two pathways, with emphasis placed on their relevance in colorectal and pancreatic cancers. We will explore how the interactions between these pathways contribute to the physiological changes displayed by cancer cells, and how these interactions are modulated throughout tumor progression and metastasis.

### The canonical Ras-MAPK pathway

**Figure 1:**
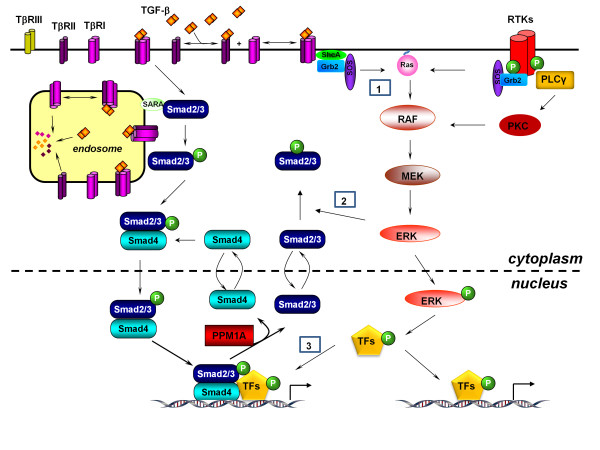
**Schematic of the Ras-MAPK and TGFβ pathways**. The left-hand side of the schematic depicts the Ras-MAPK pathway while the right-hand side of the schematic shows the Smad-dependent TGFβ signaling pathway. The details of both pathways are elaborated in the text. Both pathways begin with extracellular factor binding transmembrane cell surface receptors and end with transcriptional changes. Differences in the Ras-MAPK and TGFβ pathways are evident in the method by which stimulus signals are relayed to the nucleus. The Ras-MAPK pathway utilizes a phosphorylation cascade that results in signal amplification and a nonlinear switch-like response to stimulus. The TGFβ pathway is depicted here as a linear response to stimulus due to the direct interaction of transcription factor Smads with transmembrane receptors TGFβRI and TGFβRII. Box 1, 2 and 3 indicates points of integration that are detailed in the text.

The Ras-MAPK (Mitogen Activated Protein Kinase) pathway begins with growth factor binding to transmembrane Receptor Tyrosine Kinases (RTKs) (Figure 1). Growth factor binding initiates homodimerization and auto-phosphorylation *in trans *at specific tyrosines of the RTKs [10,11]. Growth factor receptor-bound protein 2 (Grb2) is then recruited to the cytosolic portion of the RTKs via a Src homology 2 (SH2) domain which binds to phosphotyrosines. Grb2 then recruits a guanine exchange factor, Son of Sevenless (SOS), via an SH3 domain and the GTPase Ras [10,11]. Ras, a protein that was originally identified as the transforming component in oncogenic viruses, is then post- translationally modified with an isoprenyl group that localizes it to the plasma membrane [2,12]. The GTPase activity of Ras is enhanced by the GTPase Activating Protein (GAP). Ras recruits and activates the MAPK kinase kinase (MAPKKK) Raf which initiates a phosphorylation cascade from Raf to MEK (MAPKK) which finally phosphorylates ERK (MAPK, Extracellar Regulated Kinase). Phosphorylated ERK then translocates to the nucleus where it phosphorylates transcription factors important for proliferation and differentiation [10,11].

The regulation of the activity and the specificity of MAPKs is critical for proper function and accurate transduction of extracellular signals. ERK is deactivated by MAPK phosphatases, resulting in a pathway that can terminate signaling in the absence of upstream stimuli [13]. The binding sites on ERK, auxiliary to the substrate recognition motif in the active site of ERK, confer the specificity for substrates [14]. Recent studies indicate that scaffolding proteins may play an important role in the timing and the amplitude of Ras-MAPK by regulating the location of MAPK components [15]. The scaffold either sequesters them to the cytosol and delays a response or facilitates a faster phopsphorylation cascade through the spatial concentration of the kinases (Figure 1), as has been shown for the scaffolding protein KSR (Kinase Supressor of Ras) [15,16].

### The canonical TGFβ pathway

TGF-β signals through two receptor types, the TGF-β type I and type II receptors (TβRI and TβRII, respectively) [17]. Both receptors are Ser/Thr kinases. While the type II receptor is constitutively active, the type I receptor is inactive in the absence of ligand[18]. TGF-β can bind the type II receptor independent of the type I receptor [19]. Upon binding of the ligand, TGF-β induces oligomeric receptor complex formation which enables the type II receptor to phosphorylate the type I receptor in the GS domain and consequently cause a conformational change and release of inhibitory molecules such as FKBP12[18,20]. Activated TβRI recruits and phosphorylates Smad2 and Smad3 (R-Smads), a process that is aided by SARA (Smad Anchor for Receptor Activation)[21,22]. Phosphorylation of the R-Smads causes a conformational change such that the R-Smad is released from SARA [21]. Phosphorylated R-Smad binds Smad4 forming a R-Smad/Smad4 complex, as well as forming homo-oligomeric complexes, which translocate to the nucleus and directly interact with DNA, resulting in the recruitment of coactivators or chromatin remodeling components [23]. The recruitment of coactivators, corepressors and chromatin remodeling components determines the ultimate Smad dependent cellular response to the ligand stimulation [23]. TGF-ß regulates the transcript levels of approximately 100-300 genes in various cell types (Figure 1) [1-3].

### The Non-canonical TGFβ pathway

Activation of Smads by TGF-ß is invariant in most cell types and therefore this pathway is known as the canonical TGF-ß pathway. Beside activation of Smads, TGF-ß also modulates the activity of several other signaling pathways in a ligand and receptor dependent manner (e.g. MAP kinase (MAPK) pathways, Rho-like GTPase signaling pathways, and phosphatidylinositol-3-kinase (PI3K)/AKT pathways [24-26]). Unlike the canonical pathway, modulation of these pathways by TGF-ß is often cell type specific and context dependent [27]. They are collectively referred to as non-Smad or noncanonical TGF-ß signaling pathways. Although canonical TGF-ß signaling through Smads dominated the field of TGF-ß research over the last decade, there has been increasing attention paid to the noncanonical TGF-ß signaling, particularly in the context of cell migration and epithelial-mesenchymal transition (EMT). In mammalian cells, all three MAPKs are activated by TGF-ß (Figure 1) (Each MAPK has different isoforms and, for simplicity, they are generically referred as Erk, JNK and p38). However, the kinetics of these activations vary with cell types and culture conditions [24-26,28,29]. The upstream signal transducers to MAPKs are likely small GTPases (Ras, Rac, RhoA and Cdc42) [24-26,29]. Their activities are frequently regulated by TGF-ß through diverse mechanisms. Here, we will focus on the Ras-MAPK pathways. For detailed discussions on the various noncanonical or non-Smad TGF-ß pathways, please refer to several excellent reviews on this topic [24-26,30].

It has been known for some time that TGF-ß rapidly activates Erk MAP kinases through Ras, but the magnitude of the resulting activation of Erk1/2 is much lower compared the activation by RTKs [28]. One mechanism that has been identified involves phosphorylation of ShcA by the TGFβ receptor complex on the tyrosine residues of ShcA. Similar to the RTK signaling, ShcA phoshotyrosines recruit the Grb2/SOS/Ras complex which subsequently trigger the activation of the Ras-MAPK pathway (**Box 1**, Figure 1) [31]. The biological significance of intrinsic activation of Ras-MAPK by TGFβ in normal cellular process is still poorly understood. Gene expression profiling studies implicate that TGF-β-stimulated Erk activation is involved in the modulating of a subset of genes that figure prominently in cell motility and cell-matrix interaction [32]. Some of the genes are often associated with epithelial to mesenchymal transition (EMT), an indispensable mechanism for producing mesenchymal cells, tissues and organs during normal development [33-35]. Thus, the intrinsic, relatively low, activation of Ras-MAPK is likely crucial for specific induction of genes regulating EMT and cellular motility.

### Signaling Crosstalk Between the Ras-MAPK and TGFβ pathways

The integration of Ras-MAPK and TGFβ pathways can occur via balanced activation of both canonical and noncanonical TGFβ pathways by TGFβ alone, through simultaneous stimulation of TGFβ and ligands of other signaling pathways that modulate the activity of Ras-MAPK, or through crosstalk via interactions between the intracellular effector proteins distinct to each of the two pathways. In the two lattermost mechanisms, there are two common points of integration of the Ras-MAPK and TGFβ pathways: 1) phosphorylation of coactivators of R-Smads by Erks, 2) phosphorylation of Smad2/3 by Erks in the linker region. Most of the TGFβ signaling responses are cell context dependent [27]. The cell type specific responses are in part due to Smad interaction with cell type specific transcription factors [23]. For example, activated Smad3 binds its cognate sites with Oct4 in embryonic stem cells (ESCs), MyoD in myotubes and PU.1 in pro-B cells [23]. The association between activated Smad2/3 and transcriptional cofactors can be regulated by the Ras-MAPK pathway. For example, Ras-MAPK activity has been shown to regulate the interaction of p53 and Smad2/3. In this mechanism, FGF signals through Ras-MAPK to regulate p53 phosphorylation at its N-terminus (Ser6 and Ser9) by CK1 ∈/δ [36]. Such phosphorylation of p53 enables its interaction with activated Smad2/3 to regulate target genes that are important for TGFβ cytostatic program [36]. Similarly, Src-activated Epidermal Growth Factor Receptor (EGFR) signals through Ras-MAPK to engender phosphorylation of the E-box binding transcription factor USF and facilitate its interaction with activated Smad2/3 to regulate PAI-1 gene expression [37]. Thus, the RTK activated Ras-MAPK pathway converges with the canonical TGFβ pathway at chromatin in order to regulate gene expression.

Ras-MAPK has also been shown to phosphorylate R-Smads auxiliary to the activation regions[38,39], sequestering them to the cytoplasm to attenuate TGFβ signaling [39]. However, the opposite effect of activated Ras-MAPK on Smad activity has also been reported [40-42]. A possible explanation for the contradictory observations is that the levels of Ras activity could dictate the outcome of the experiment [43]. In a majority of human cancers carrying oncogenic Ras mutations, the activity rather than the levels of Ras expression is elevated. Thus, the levels of Ras activity translate into the levels of MAPK activity, ultimately shaping the TGFβ response via crosstalk between R-Smads and the Ras-MAPK pathway.

The linker region between Mad Homology Domains 1 and 2 (MH1 and MH2) have been shown to be phosphorylated by GSK-3 and ERK1/2. Phosphorylation of Ser204 on Smad3 via GSK-3 kinase activity results in decreased affinity between Smad3 and CREB binding protein, suggesting that the functional significance of the linker region phosphorylation status is to modulate transcription factor activity of SMAD proteins, rather than to regulate the nucleocytoplasmic localization of Smad proteins[44]. Consistent with this theory, the linker region phosphorylation of Smad2 and Smad3 by ERK1/2 at positions Ser240/Ser245/Ser250 and Ser204/Ser208/Ser215, respectively, has been shown to enhance Smad transcriptional activity of the Type I and Type III Collagen gene [40,45]. Additionally, Small C-terminal Phosphatase (SCPs) in the nucleus have been shown to dephosphorylate the linker region phosphorylation sites in Smad2, confirming a primarily nuclear role of the linker region phosphorylation [46]. Such dephosphorylation by SCPs results in activation of Smad dependent transcriptional regulation of exogenous luciferase reporters[46]. Thus, conflicting results about the functional consequences of linker region phosphorylation suggest that not only is Smad2/3 transcriptional activity regulated in a cell type specific manner, but also perhaps in a single cell type discrete genes are positively or negatively regulated by linker region phosphorylation in a chromatin context dependent manner.

Taken together, the current and previously reported data strongly suggests that different cellular responses to TGFβ stimulation in different cell types and cellular contexts largely results from the status of MAPK activity at the time of ligand stimulation.

### Systems Features of MAPK and TGFβ pathways

Even though the signaling of the TGFβ and Ras-MAPK pathways initiates upon ligand binding at the plasma membrane, the modes of signal transmission are quite different [47,48]. The Ras-MAPK pathway features a cascade of sequential kinase phosphorylations/activations from RAF to ERK (Figure 1). This type of pathway architecture can lead to significant amplification of the original upstream receptor/ligand binding signal. The RTKs also activate other signaling pathways such as PLCγ, PI3K or Src. Some of these signaling pathways can feed into Ras-MAPK and produce positive feedback loops. There are also multiple well characterized negative feedback loops in the Ras-MAPK pathway that cause desensitization or dampening of the signal [49]. The presence of these feedback loops have been shown to produce switch-like (i.e. bistable) responses to receptor activation, allowing for a tight threshold of cellular response during development [50,51].

Unlike the Ras-MAPK pathway, the TGFβ signal relay system is relatively short. There are no apparent signal amplification steps in the signaling cascade subsequent to the receptor phosphorylation of R-Smads. The constant shuttling of Smads in and out of the nucleus allows the Smads to constantly monitor receptor activity [48]. This type of arrangement results in a more linear response to ligand binding. However, a more recent study reveals that TGFβ depletion during signaling is important to produce threshold-like input/output response to receptor activation [52]. Clearly, there is also nonlinearity in the TGFβ pathway.

Systems analysis of biological signaling pathways suggests that signaling pathways interact with one another and the final biological response is shaped by interaction between pathways [51]. Since there are multiple integration points between the Ras-MAPK and TGFβ pathways, it is not a surprise that the integration of these two pathways produces quite complex biological outcomes, depending on the cellular context. The challenge is how to precisely determine the behaviors of the interacting pathways and how to manipulate the end-point biological responses in normal and cancer cells.

### Lesions in the Ras-MAPK and TGFβ pathways in Cancers

Colorectal and pancreatic cancers are, respectively, the fourth and fifth causes of cancer deaths [53]. More than one third of colorectal cancer patients develop metastasis. Of these cancers, about 35% are the result of genetic predisposition due to inherited lesions in the TGFβ pathway [54]. Additionally, mutations that inactivate the key signal components, including receptors and Smads, are frequently observed within the tumor. Loss of function mutations in TβRII have been found in a majority of colorectal and gastric carcinomas with microsatellite instability (MSI) [55,56]. In microsatellite stable colon cancer cell lines, missense mutations are identified in ~15% of cases [57]. Inactivating mutations of TβRI occurs at low frequency in pancreatic and biliary carcinomas [58] but relatively high in ovarian cancers with wild type *TβRII *[59]. In one example of TGFβ pathway lesion, TGFβRI*6A, the TGFβRI is missing three alanines from the signal sequence. TGFβRI*6A was previously associated with hereditary cancers based on analysis of a limited numbers of cases [60,61]. Recent work using a much larger number of case studies suggests that there is no increased hereditary risk of colon cancer associated with this mutation [62]. However, it is still possible that TGFβRI*6A does convey increased risk in specific patient populations or in somatic mutations [62].

Inactivation of TGF-β signaling also occurs by disabling Smad proteins. *SMAD4 *was originally identified as a tumor-suppressor gene lost in pancreatic cancers called DPC4 (deleted in pancreatic cancer 4) [63]. Subsequent studies revealed that deletion of Smad4 also occurs in 16-25% of colorectal cancer [64]. Germline mutations of *SMAD4 *are associated with juvenile polyposis and hamartomatous polyposis [65,66]. Inactivating mutations of Smad2 have been identified in ~6% of colorectal cancers while loss of function mutations of Smad3 are rare in colorectal cancers [64,67].

Escaping the TGFβ growth constraint is one of the hallmarks of tumor cells [2]. Tumor cells explore different mechanisms to achieve this. Despite the fact that inactivating mutations in the canonical TGFβ pathways in colorectal and pancreatic tumors are wide spread, cells isolated from other tumor types preserve the functionality of the canonical TGFβ signaling components and achieve TGFβ growth resistance by altering other aspects of the pathways [5]. Thus, oncogenic activation can also interdict TGFβ growth inhibitory responses by modifying the activity of downstream elements such as cell cycle inhibitors or transcriptional cofactors of Smad transcription factor activity.

Throughout tumor progression, the TGFβ ligand becomes highly expressed and actually promotes proliferation, particularly in the case of prostate cancer [68]. This may be due to its ability to stimulate MAPK pathways [69]. High levels of TGFβ are correlated with increased angiogenesis, as the tumor recruits blood vessels to allow for greater growth by inducing greater TGFβ dependent VEGF expression [70]. In the clinic, patients with high levels of TGFβ have decreased survival, likely due to aggressive tumor progression and metastasis [71]. In addition to over secretion of the TGFβ ligand, deregulation of the intracellular pathway can sensitize cells to an unchanging amount of TGFβ ligand. For example, *in situ *cell culture RNAi knockdown of Phosphatase and Tensin Homolog (PTEN), a TGFβR phosphatase, resulted in the increased TGFβ dependent invasion potential of cells [72]. Recent studies also suggest that TGFβ is involved, in some cell types, in directing metastatic cells to specific locations such as bone [73,74]. The cellular response to TGFβ in tumors clearly plays a role in tumor metastasis, even though there is not strong evidence that R-Smads mediate this role.

**Figure 2:**
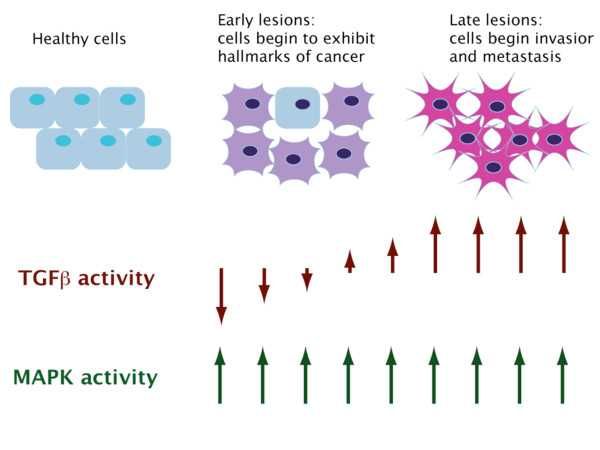
**Schematic showing the transitions from healthy to metastatic cells and the corresponding changes to TGFβ and MAPK signaling**.

The Ras-MAPK pathway is an important component of many cancerous cells. The majority of cancer associated lesions to the Ras-MAPK pathway result in constitutive activation of the pathway [12]. Mutations that have been identified are located early in the pathway and include overexpression of RTKs, activating mutations to RTKs, sustained expression of activating autocrine or paracrine ligands, and mutations to Ras and Raf [2,12]. Temporally, these lesions occur early in tumorigenesis and are sustained for the life of the cancer (Figure 2). Ras mutations have been found in 30% of all cancers, 90% of pancreatic cancers and 50% of colon cancers [12]. B-Raf mutations have been found in 65% of melanomas, 45% of papillary thyroid cancers and 36% of ovarian cancer [75-77]. In summary, a large portion of human tumors have a constitutively active Ras-MAPK pathway and have acquired resistance to TGFβ induced cell cycle arrest. Thus, the role of TGFβ in cancer progression seems to be largely through lesions that stimulate the non-cannonical signaling, and are aided by inactivating lesions in the canonical pathway, which are associated with cell-cycle arrest and the apoptotic response.

### Crosstalk between TGFβ and Ras-MAPK Signaling Pathways drives EMT

Crosstalk between the TGFβ and Ras-MAPK pathways appears to be required for tumor metastasis, likely through the role of crosstalk in epithelial-mesenchymal transition (EMT). TGFβ induced EMT, which is a fundamental mechanism that drives metastasis *in vivo *or invasion *in vitro*, requires constant TGFβ signaling to become a stable phenotype [78,79]. The connection between EMT and metastasis is exemplified through the finding that fibroblast cells that undergo EMT are more conducive to promote tumor invasion [80]. EMT is marked by changes to the actin cytoskeleton, loss of cell polarity, and migration/invasion [35,81]. Acting alone, neither pathway is successful in permanently converting from an epithelial to a mesenchymal phenotype [41]. Long-term expression and cooperation of TGFβ and Ras- MAPK causes full EMT through induction of cytokine feedback loops that include upregulating TGFβ autocrine signaling [78]. Recently, a new mechanism by which TGFβ induces EMT has been introduced, where TGFβ induces isoform switching of fibroblast growth factor (FGF) receptors [80]. Prolonged treatment of TGFβ results in increased FGFR1IIIc, which is the mesenchymal isoform, and decreased expression of the epithelial isoforms of FGF receptors. FGF-2 but not FGF-7 signals through FGFR1IIIc[80]. The switch in FGF-2 receptor expression enables cells to respond to FGF-2 to activate Ras-MAPK. Furthermore, epithelial cells treated with both TGFβ and FGF-2 undergo EMT instead of epithelial to epithelial-myofibroblastic transition (EMyoT). Thus, the crosstalk between the Ras-MAPK and TGFβ pathways may be harnessed to promote tumor growth through EMT.

## Conclusions and Future Directions

Colorectal and pancreatic cancers account for a large portion of cancer incidence and fatalities in the United States. Thus, they are important models for the study of the complicated roles of Ras-MAPK and TGFβ pathways. As more is learned about these pathways in normal and cancerous cells, it becomes increasingly clear that crosstalk between the distinct pathway components are important for TGFβ signal interpretation in a cell type and cell context dependent manner. Future studies are required to address the interdependency of these pathways, paying close attention to the connection between the cellular context in a study, the level of contribution of the Ras-MAPK pathway to the TGFβ response, and the ultimate cellular response to TGFβ ligand stimulation.

A consequence of this emerging theme of crosstalk may be the reevaluation of cancer therapeutic strategies. Currently, Ras and Raf have been the hot targets in the Ras-MAPK pathway for anticancer drugs. Inhibition of isoprenylation of Ras seemed promising. However, it appears that the enzymes that catalyze this reaction are somewhat promiscuous and off-target effects have limited the successful development of inhibitors of Ras. Other Ras-MAPK components such as Raf, MEK and ERK are also being investigated and are proving more successful drug targets [82]. One drug targeting Raf has been approved by the FDA for treatment of renal cell carcinoma and a handful of other drugs are in Phase I/II/III trials that target Raf and MEK [82]. TGFβ's involvement in promoting a metastatic phenotype in aggressive cancers makes it a highly sought after pathway to target for anticancer therapies. The obvious targets of choice are the TGFβ ligand itself, the ligand- binding surface on TGFβRI/II and betaglycan [83]. As a result, a humanized anti- TGFβ monoclonal antibody (GC1008) is in Phase I/II clinical trials. Additionally, soluble TGFβRII and betaglycan recombinant receptors, which compete for extracellular TGFβ, as well as inhibitors TGFβRI kinase activity have shown encouraging anti-metastatic results [84]. As might be the case for many kinase inhibitors, long term administration of a single agent may select for more aggressive drug resistant tumor variants, as has been demonstrated for LY2109761, a TβRI/TβRII kinase inhibitor in a mouse skin model [85]. Considering the synergistic effects of TGFβ and MAPK pathways in tumorigenesis it is logical to try a combinatorial approach in cancer treatments, where simultaneous partial inhibition of the Ras-MAPK and TGFβ pathways, yields synergistic inhibition of TGFβ ligand induced metatstasis, while having minimal impact on other aspects of TGFβ and Ras-MAPK biology.

## Competing interests

The authors declare that they have no competing interests.

## Authors' contributions

DAC and XL wrote and edited the article. DAC and XL designed Figure 1. LW, JB and TR designed Figure 2 and wrote the initial summary and portions of the article. DAC and XL compile the final references. All authors read and approved the final manuscript
